# Robotic Approach to Multivisceral Resection for Gastrointestinal Stromal Tumor With En Bloc Resection of the Stomach, Distal Pancreas, and Spleen: A Case Report

**DOI:** 10.7759/cureus.96479

**Published:** 2025-11-10

**Authors:** Justin M Bader, Sean Liu, Kevin Billingsley

**Affiliations:** 1 Surgical Oncology, Yale New Haven Hospital, New Haven, USA; 2 Surgical Oncology, National Cancer Institute of the National Institutes of Health, Bethesda, USA; 3 Surgical Oncology, Yale University, New Haven, USA; 4 Surgical Oncology/Surgery, Yale University, New Haven, USA

**Keywords:** complex surgical resection, gastrointestinal stromal tumor, interdisciplinary cancer treatment, robotic-assisted surgery, transition in operative approach

## Abstract

Gastrointestinal stromal tumors (GISTs) are rare mesenchymal neoplasms of the gastrointestinal tract. While surgical resection is standard for GISTs greater than 2 cm in diameter or those with high-risk features, extraluminal growth and invasion of adjacent organs can present an intraoperative challenge. We report the case of a 75-year-old woman with a large GIST and an unexpected intraoperative finding of direct invasion into the pancreas and spleen, requiring partial gastrectomy with en bloc distal pancreatectomy and splenectomy. A tailored operative approach with a skilled bedside assistant allowed for a successful, complete resection of the GIST and an uncomplicated postoperative recovery. Adjuvant imatinib was started per discussion with the interdisciplinary tumor board. This case highlights the importance of intraoperative flexibility and team-based conduct in managing large GISTs.

## Introduction

Although rare overall, gastrointestinal stromal tumors (GISTs) are the most common type of mesenchymal tumor found in the gastrointestinal tract, with an incidence of 10-15 cases per one million people [1]. As 10-30% of lesions progress to malignancy, GISTs with high-risk features or a size greater than 2 cm are usually considered for surgical resection [2,3]. However, 37-56% of GISTs exhibit extraluminal growth, a feature of this rare tumor that can complicate surgery [4,5]. This case report describes a unique and unexpected intraoperative finding of a GIST directly invading the pancreas and spleen, which was successfully managed with a transition to a multivisceral resection.

## Case presentation

**Figure 1:**
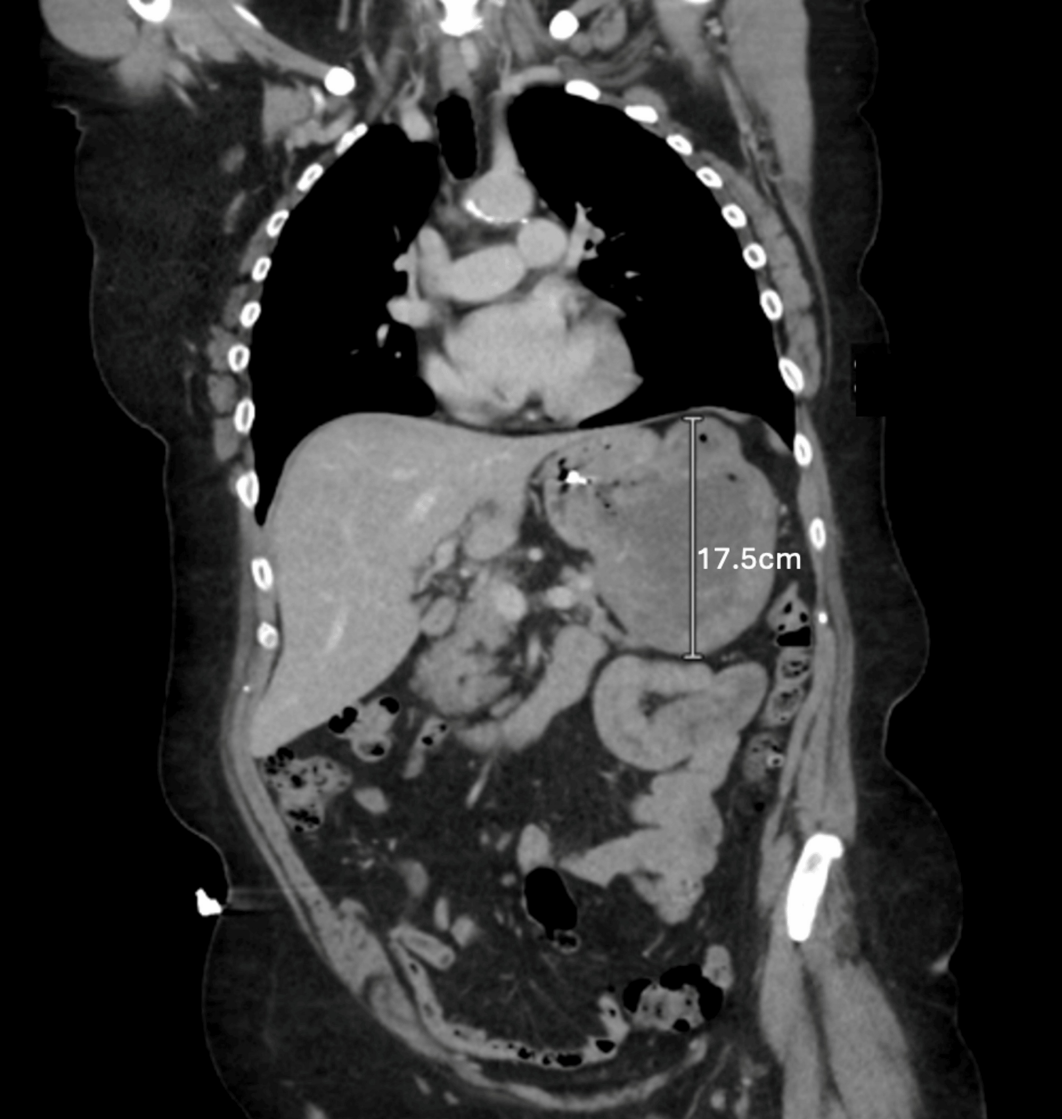
Abdominal CT scan with IV contrast visualizing a large exophytic gastric mass.

A 75-year-old patient with well-controlled hypertension, obesity (BMI 38), and no prior abdominal surgeries presented with chronic, persistent gastroesophageal reflux unresponsive to an extended course of acid-suppressing medications. On physical examination, the patient had a symmetric, non-tender, non-distended abdomen with no bulges or obvious abnormalities. Diagnostic upper endoscopy by the patient’s gastroenterologist revealed a friable, hemorrhagic mass involving the greater curvature of the stomach, with endoscopic biopsies showing a C-kit-positive GIST without any mitoses in 25 high-power fields. An abdominal CT scan with IV contrast confirmed a large, exophytic mass, approximately 10.1 × 12.0 cm in diameter (Figure 1), interposed between the pancreatic tail and the posterior wall of the midbody of the stomach, with no evidence of pancreatic invasion or metastatic disease. The patient was referred to the surgical oncology service, where it was decided that the patient would undergo a partial robotic gastrectomy for GIST resection.

**Figure 2:**
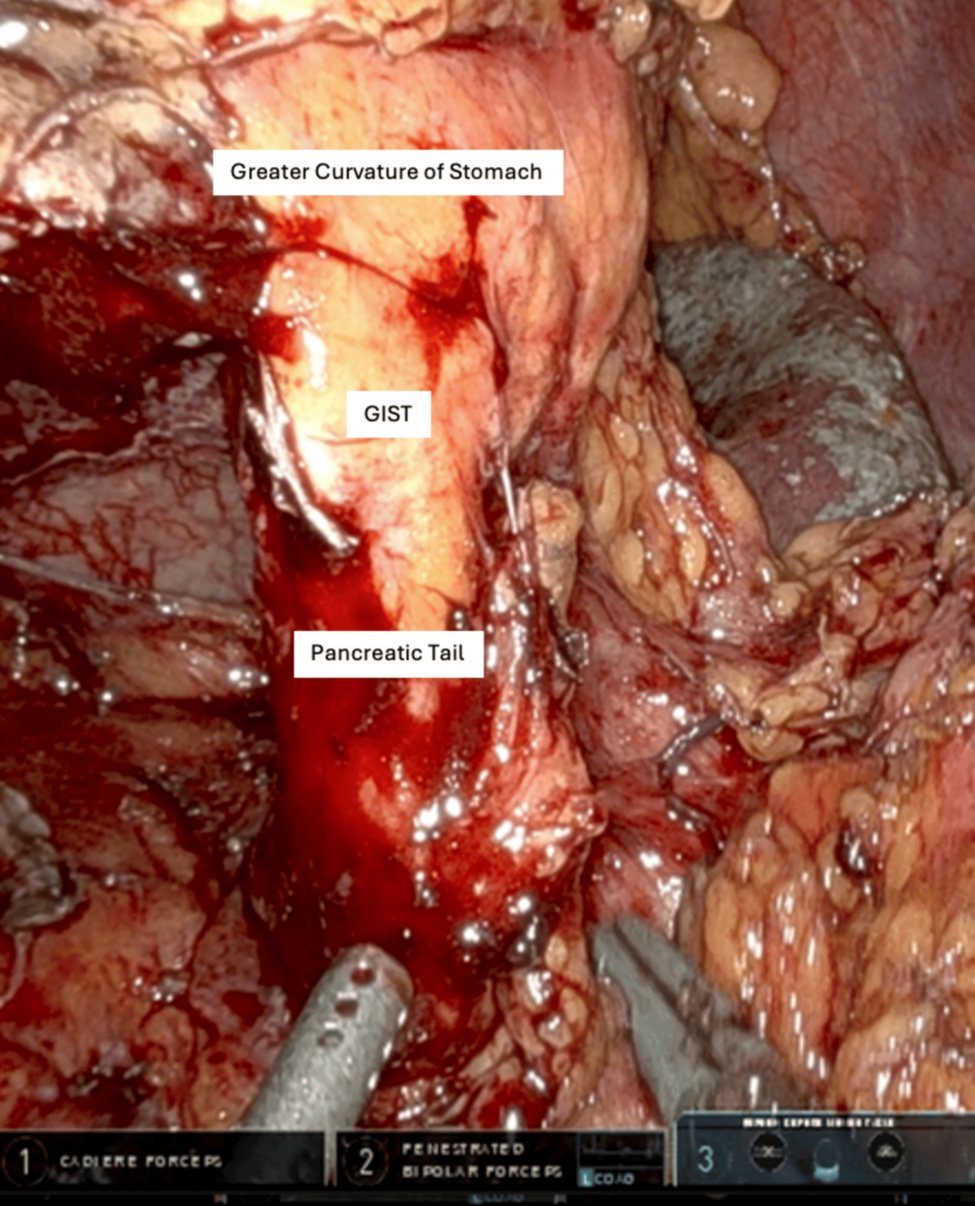
Picture showing intraoperative view of GIST adherent to pancreatic tail. GIST: gastrointestinal stromal tumor.

In the operating room, the lesser sac was opened, and a large submucosal mass was seen on the posterior wall along the greater curvature of the stomach. Dissection of the mass and mobilization of the greater curvature were attempted, but it became evident that the mass was adherent to and possibly involving the spleen and pancreatic tail (Figure 2). After encountering difficulty in dissecting the pancreas from the tumor, the decision was made to perform an en bloc distal pancreatectomy and splenectomy to achieve complete resection of the GIST.

Peripancreatic dissection was performed, and the splenic artery and vein were identified, ligated with 2-0 silk ties and hemoclips, and then divided using robotic scissors (Video 1). The pancreas was circumferentially mobilized and encircled with a surgical umbilical tape to allow retraction and optimal visualization. The pancreatic midbody was divided by the bedside assistant through an assist port using a 60 mm tri-stapler with varying staple heights and a reinforcing strip (Video 2).

**Video 1:** Intraoperative view of robotic-assisted splenic artery ligation. Meticulous dissection and ligation of the artery with suture ligation and clip placement ensured no leakage.

**Video 2:** Intraoperative view of robotic-assisted pancreatic transection. Pancreatic transection with felt re-enforced stapler through the assist port allowed for ideal visualization and successful transection of the pancreas without leakage.

To visualize the extent of the GIST and ensure proper resection margins, a deliberate gastrotomy of approximately 3 cm was made along the greater curvature of the stomach bordering the GIST. With intraluminal visualization of the tumor, the gastrotomy was extended posteriorly along the stomach wall adjacent to the GIST margin using a robotic vessel sealer (Video 3). After resection of the GIST from the stomach wall, the resulting 10 cm gastrotomy was approximated for closure with interrupted 2-0 Vicryl sutures (Video 4). The suture tails were retracted anteriorly to ensure complete visualization of the gastrotomy plane, and a robotic stapler was then used to divide the stomach just posterior to the established suture line (Video 5).

**Video 3:** Intraoperative view of robotic-assisted tumor resection from stomach body. Large GIST must be properly visualized during resection to ensure proper resection margins while also preserving as much healthy stomach tissue as able. GIST: gastrointestinal stromal tumor.

**Video 4:** Intraoperative view of robotic-assisted approximation of gastrotomy closure with sutures. Complex closure of the large gastrotomy was conducted with extreme precision with suture approximation for proper gastrotomy closure alignment.

**Video 5:** Intraoperative view of robotic-assisted gastrotomy closure using a robotic stapler. After correct alignment of the gastrotomy with sutures, the stapler allows for a clean transection with tight seal to avoid postoperative leaks.

The GIST was removed from the abdomen en bloc with the partial gastrectomy and distal pancreatectomy using a specimen retrieval bag. The spleen was subsequently removed along with the surrounding gastric wall margin. A 19 French Blake drain was placed in the lesser sac, posterior to the stomach and adjacent to the pancreatic remnant. Robotic port sites were closed with 4-0 Monocryl sutures, after which the patient was extubated and brought to the postoperative care area. The estimated blood loss was 700 cc, and no blood product transfusions were required. After being discharged home on postoperative day 3, the patient was presented at a multidisciplinary tumor board and subsequently started on a one-year course of imatinib.

Pathologic examination of the resected specimen showed a 12.5 cm low-grade GIST, spindle cell type, with a mitotic rate of one mitosis per 5 mm² and negative resection margins. Of the 23 lymph nodes examined, none were positive for disease, and the pathologic stage was stage II (pT4 N0). Upon clinical follow-up, the patient had no reported postoperative complications. Moreover, an abdominal and pelvic CT scan taken four months after resection showed no evidence of recurrent disease.

## Discussion

GISTs most commonly arise within the stomach but may demonstrate extraluminal growth and invasion of adjacent organs [4,5]. The case presented here underscores the need for not only meticulous preoperative planning but also intraoperative adaptability. While preoperative imaging aids in defining tumor extent, the surgeon must remain prepared for unexpected anatomy and, when necessary, multivisceral resection.

Standard practice is to resect all GISTs 2 cm in diameter, although operative criteria vary by institution. In most cases, GIST resection can be accomplished through a simple gastric wedge resection using an open, laparoscopic, or robotic approach, depending on the provider’s expertise and available resources. Additionally, GISTs can often be identified by upper endoscopy and do not require a biopsy, as there is a small risk of hemorrhage within the tumor during the biopsy procedure. However, in certain cases, such as large GISTs or concerns for metastatic disease, a biopsy may be helpful for prognostication and determining eligibility for neoadjuvant imatinib to downsize the tumor before resection. In the discussed case, the GIST appeared to be large but without radiographic evidence of invasion and zero mitoses per 25 high-power fields on upper endoscopy biopsy. For these reasons, a simple gastric resection without neoadjuvant imatinib was deemed the appropriate preoperative choice.

In this case, GIST invasion of the pancreas and spleen was discovered intraoperatively, and additional resection was necessary to achieve complete tumor clearance. For example, an umbilical tape encircled around the pancreas allowed for retraction of the bulky GIST and provided optimal visualization during distal pancreatectomy. Furthermore, a GIST of this size can make it difficult to determine adequate resection margins. Innovative maneuvers, such as creating a deliberate gastrotomy adjacent to the GIST, allowed for clear visualization of the depth of intraluminal involvement. This approach ensured adequate resection margins and a successful operation.

Robotic platforms have shown advantages in GIST resection, especially in anatomically challenging gastric sites. Enhanced optics and magnification support precise dissection, while articulating instruments provide improved range of motion for complex gastric resections. In one series of 25 consecutive patients with GIST, robotic-assisted subtotal gastrectomy achieved negative margins, minimal blood loss, and median hospital stays of three days or less, without conversion to open surgery in any case [6]. Previous studies have also found that minimally invasive approaches yield shorter hospital stays and lower perioperative mortality, in addition to comparable oncologic outcomes to open surgery for GIST resection [7-9].

This case also highlights the critical value of a skilled bedside assistant in the success of robotic-assisted complex GIST resection. Notably, an assist port may provide a better angle for stapler division of the pancreatic body, playing a vital role in providing optimal tissue retraction and exposure during key moments of the operation. Additionally, multidisciplinary collaboration remains central to GIST management. Pathologic and molecular features guide decisions regarding adjuvant tyrosine kinase inhibitor (TKI) therapy, particularly imatinib. Early engagement with oncology teams supports risk stratification and treatment planning.

## Conclusions

In summary, this case highlights extraluminal involvement of a GIST with direct invasion into the pancreas and spleen, which was discovered intraoperatively. Despite the unexpected findings, the tumor was successfully managed with partial gastrectomy and en bloc distal pancreatectomy and splenectomy. The operative strategy, which incorporated effective assistant port usage and close intra- and postoperative team coordination, demonstrates how extended multivisceral resection can be successfully performed using a robotic platform to avoid a significant abdominal incision and achieve favorable oncologic and postoperative outcomes.
